# Knowledge, Attitudes, Practices, and Associated Factors of Healthcare Workers Toward Hepatitis B Post‐Exposure Prophylaxis in Ethiopia: A Cross‐Sectional Study

**DOI:** 10.1002/hsr2.72123

**Published:** 2026-03-19

**Authors:** Esubalew Muluneh Aligaz, Zekarias Markos, Sitotaw Tesfa Zegeye, Samuel Debas Bayable

**Affiliations:** ^1^ Department of Anesthesia Bahir‐Dar University College of Medicine and Health Science Bahir‐Dar Ethiopia; ^2^ Department of Anesthesia Wachemo University College of Medicine and Health Science Hossana Ethiopia

**Keywords:** Ethiopia, healthcare workers, Hepatitis B virus, knowledge, attitude, and practice, post‐exposure prophylaxis, practice

## Abstract

**Background:**

Hepatitis B virus (HBV) infection remains a major occupational hazard for healthcare workers (HCWs), particularly in low‐resource settings. Timely and appropriate post‐exposure prophylaxis (PEP) is essential to prevent HBV transmission following occupational exposure; however, its effective use depends largely on healthcare workers’ knowledge, attitudes, and practices (KAP).

**Objective:**

To assess the knowledge, attitudes, and practices of healthcare workers regarding HBV post‐exposure prophylaxis and to identify factors associated with adequate knowledge.

**Methods:**

A hospital‐based cross‐sectional study was conducted from January to March 2025 at Wachemo University Nigist Eleni Mohammed Memorial Comprehensive Hospital, Ethiopia. Using stratified and systematic random sampling, 422 HCWs were selected. Data were collected using a structured self‐administered questionnaire and analyzed with SPSS version 26. Bivariable and multivariable logistic regression analyses were performed to identify factors associated with adequate knowledge. Adjusted odds ratios (AORs) with 95% confidence intervals (CIs) were calculated, and *p* ≤ 0.05 was considered statistically significant.

**Results:**

Of the 422 eligible HCWs, 400 participated (response rate: 94.8%). Only 32.5% demonstrated adequate knowledge of HBV PEP, although 70.0% had a positive attitude. HBV vaccination coverage and PEP utilization following occupational exposure were low. Factors independently associated with adequate knowledge included prior HBV‐related training (AOR = 2.10; 95% CI: 1.20–3.90), history of occupational exposure (AOR = 3.72; 95% CI: 2.38–5.80), positive attitude toward PEP (AOR = 3.35; 95% CI: 1.95–5.78), and HBV vaccination status (AOR = 1.07; 95% CI: 1.01–2.86).

**Conclusions:**

Despite positive attitudes, healthcare workers demonstrated substantial gaps in knowledge and practice of HBV PEP, leading to underutilization of services; strengthening training, vaccination coverage, and institutional PEP guidelines is essential to improve occupational safety in low‐resource settings.

**Abbreviations**
HCWs: Health care workers
HBV: Hepatitis B Virus
HBC: Hepatitis C virus
KAP: Knowledge, attitude, and practice
PEP: Post‐exposure prophylaxis
WHO: World Health Organization

## 1 Background

Hepatitis B virus (HBV) is a life‐threatening liver infection classified under the Hepadnaviridae family [1]. Hepatitis B virus (HBV) infection remains a significant global health issue, with serological evidence indicating that 30% of the world's population has experienced either recent or past infection [2]. Approximately 1.5 million new HBV cases are reported annually [3]. Furthermore, around 240 million people worldwide are chronic carriers of HBV [4]. The scale of this issue is considerably larger in developing nations across Asia and Africa [5]. Chronic infection of HBV increases the risk of liver cirrhosis, hepatic decompensation, or hepatocellular carcinoma [6].

HBV Hepatitis B is a communicable disease [7], transmitted from person to person through exposure to infected blood or bodily fluids [8]. Therefore, exposure to any of these body fluids increases the risk of acquiring the virus [9]. Healthcare workers, handling sharps and having needle stick injuries (NSI), are at elevated risk since they may be exposed to multiple infections, including HBV [10]. The risk of HBV infection in health care workers is 3–5 times higher than in the general population [11]. Each year, around 3 million health care workers (HCWs) experience occupational exposure to HBV [8].

Currently, there is no effective treatment for HBV. The most effective and practical way to prevent HBV transmission between healthcare workers (HCWs) and their patients is through Hepatitis B vaccination [12]. The vaccine offers 90–100% protection against infection [13]. The hepatitis B vaccine also ensures long‐term, and possibly lifelong, immunity in those who receive all recommended doses [14]. In addition, adopting universal precautions, such as protective barriers (like gloves), strict sterilization protocols, proper medical waste disposal, and vaccination, helps prevent HBV infection [15, 16]. However, the effectiveness of the HBV vaccine is highly affected by the knowledge, attitude, and practice of health care workers [17]. Furthermore, the health‐related behavior of healthcare workers would affect the effectiveness of the prevention of this virus [18]. Therefore, this study aims to assess the knowledge, attitudes, and practices of healthcare workers regarding post‐exposure prophylaxis for HBV.

## 2 Objective

The primary end point of this study was to assess the knowledge, attitudes, and practices of healthcare workers on post‐exposure prophylaxis for the hepatitis B virus.

## 3 Methods

### 3.1 Study Design, Period, and Area

A hospital‐based cross‐sectional study was conducted at Wachemo University Nigist Eleni Mohammed Memorial Comprehensive Hospital, which is the main referral center for patients in central Ethiopia, from January to March 30, 2025. These places were selected because they are tertiary healthcare facilities and public health centers that offer specialized clinical inpatient and outpatient services to a large portion of the central Ethiopian population. The total workforce at this hospital comprises 802 professionals, including nurses, midwives, physicians, laboratory technicians, and anesthetists.

### 3.2 Study Population, Sampling Size Determination, and Sampling Technique

**Figure 1:**
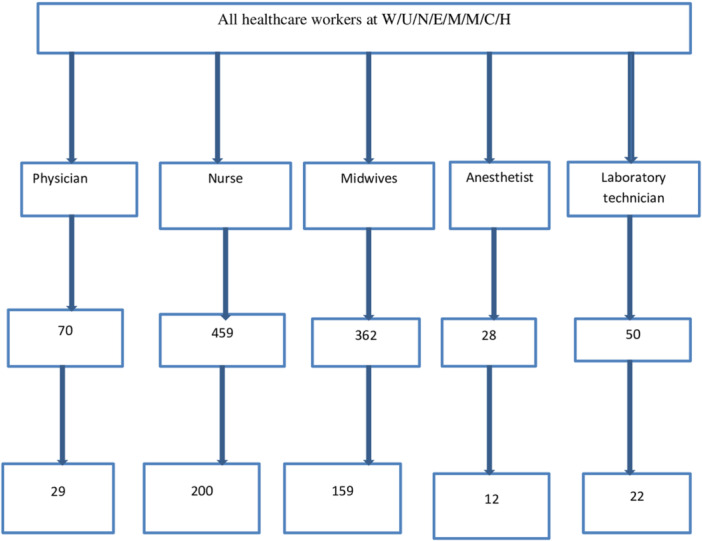
The flow chart of study participant selection from each stratum.

The study population included all healthcare professionals working at Wachemo University Nigist Eleni Mohammed Memorial Comprehensive Hospital during the study period, including physicians, medical laboratory technicians, nurses, midwives, and anesthetists. The sample size was determined using a 50% prevalence because we have not found the literature about the assessment of knowledge, attitude, and practice towards post‐exposure prophylaxis of hepatitis B virus among healthcare workers in Ethiopia. The sample size was determined using a single proportion formula: *n* (*z*_α/2)_^2^p (1‐*p*)/*d* ^2^ Where *n* minimum sample size, *p* = an estimated prevalence rate for the population, *d* = the margin of error (0.05), and at a 95% confidence interval, where *z*_α/2_ = 1.96, *p* = the proportion of knowledge, attitude practice of PEP HBV (50%). There is no similar study in the study area. The study's final sample size was 422, considering a 10% non‐response rate. A stratified sampling technique was used to create strata for different professionals, and a separate sample was taken by proportion to allocate the health workers based on their numbers independently from each stratum by using a systematic random sampling technique to select study subjects among health care professionals who are working at Wachamo University, Nigist Eleni Mohammed Memorial Comprehensive Hospital. The size of each stratum was determined based on the proportion of each profession in the total workforce, and the sample within each stratum was selected using a systematic random sampling technique with a calculated sampling interval *k* derived from *N*/*n*. The first participant in each stratum was selected randomly, and every *k* ^th^ person thereafter was included **(**Figure 1 **)**.

### 3.3 Operational Definitions

**Knowledge**

1. Good knowledge: If respondents correctly answered ≥ 70% of the knowledge items [15, 19].

2. Poor knowledge: If respondents answered < 70% of the knowledge items [15, 19].

**Attitude**

1. Positive attitude: If participants correctly answered ≥ 70% of the attitude items [15, 19].

2 Negative attitude: If participants answered < 70% of the attitude items [15, 19].

**Practice**

1. Good practice: If participants correctly answered ≥ 70% of the practice items [15, 19].

2. Poor practice: If participants answered < 70% of the practice items [15, 19].

### 3.4 Data Collection Tool and Procedure

Data were collected by using a structured questionnaire. The questionnaire included written consent, sociodemographic variables, knowledge, attitude, and practice questions towards PEP for the hepatitis B virus, which were developed by adapting from different peer‐reviewed literature [20, 21, 22]. Data were collected face‐to‐face using a structured questionnaire, which was distributed and retrieved by trained data facilitators in person to ensure completeness, privacy, and confidentiality. To minimize self‐reporting and recall biases, the questionnaire was completed anonymously, participants were assured of confidentiality, and data collectors received training to standardize instructions and avoid leading questions.

### 3.5 Scoring of Knowledge, Attitude, and Practice

In this study, knowledge was assessed using nine equally weighted questions, scored as “1” for correct and “0” for incorrect responses. Participants who correctly answered at least seven questions (≥ 70%) were classified as having adequate knowledge of PEP for HBV. Attitude was evaluated using seven questions, with those scoring ≥ 70% (at least five correct responses) categorized as having a positive attitude. Practice was assessed through seven questions. Participants who provided correct responses to more than ≥ 70% (at least five correct responses) were considered to have good practice. The questionnaire was prepared with “Yes” for correct and “No” for incorrect responses. The ≥ 70% threshold is consistent with previous KAP studies conducted in Ethiopia and other low‐resource settings.

### 3.6 Data Quality Control

Data quality was ensured through pretesting of 5% of the questionnaires before data collection, with internal consistency assessed using Cronbach's alpha (*α* = 0.77). Data completeness and consistency were independently verified by two professionals in the relevant field. Data collectors and supervisors received adequate training, and daily supervision was conducted to identify and resolve incomplete questionnaires. All statistical tests were two‐sided, with a significance level of *p* ≤ 0.05. Statistical analyses were performed in accordance with the SAMPL reporting guidelines.

### 3.7 Data Processing and Statistical Analysis

The collected data were checked for completeness before data entry, and records with missing data were excluded. The cleaned dataset was then imported into SPSS version 26 for analysis. The data was summarized by statistical analysis for descriptive statistics of the variables. For categorical variables, frequencies and percentages were calculated. The bivariate and multivariate logistic regression model was used for the assessment of associated factors linked to the independent variables and the results. We computed odds ratios (OR) with 95% confidence intervals (95% CI). The multivariate logistic regression model for associated factor analysis incorporated all variables with a *p* value ≤ 0.25 (to control the influence of confounding) in the bivariate analysis. This allowed for the calculation of the adjusted odds ratio (AOR) with 95% confidence intervals. The Hosmer and Lemeshow test was used to assess the final binary logistic regression model's fitness at a *p* value > 0.05. A p‐value of ≤ 0.05 was considered statistically significant in all analyses.

## 4 Results

### 4.1 Socio‐Demographic Characteristics of HCWs

Of the 422 eligible participants, 400 healthcare workers with different professions participated in the study, with a response rate of 94.8%. Among them, 167 (41.8%) were male, and 233 (58.2%) were female. The majority were aged 30–39 years (255, 56.3%), with a mean age of 32 years. In terms of years of service, 255 (63.7%) had worked for 5–10 years, 132 (33.0%) for 6 months to 4 years, and 11 (3.3%) for more than 10 years. Most participants were nurses (48.3%) and midwives (37.5%) (Table 1).

**Table 1:** Socio‐demographic characteristics of HCWS at Wachemo University Nigist Eleni Mohammed Memorial Comprehensive Hospital.

Variables	Number (*N*)	Frequency (*f*)
**Sex**		
Male	167	41.8%
Female	233	58.2%
**Age in years**		
20–30	165	41.3%
31–40	255	56.3%
41–50	10	2.5%
51–60	0	
**Marital status**		
Single	269	67.3%
Married	123	30.8%
Separated	4	1%
Divorced	4	1%
**Profession**		
Physician	26	6.5%
Nurse	193	48.3%
Laboratory	20	5%
Anesthetist	11	2.8%
Midwifery	150	37.5%
**Level of education**		
Diploma	29	7.2%
BSC	284	71%
MSC	60	15
Physician	27	6.8%
**Work experience**		
6 months to 4 years	132	33%
5–10years	255	63.7%
> 10 years	13	3.3%
**Monthly income**		
> 5000	28	7%
5000–10000	238	59.5%
> 10000	134	33.5

*Note: N* = 400 (denominator used to calculate the percentages). BSc = Bachelor of Science, HBV=hepatitis B virus, MSc = Master of Science

### 4.2 Knowledge Level of HCWs about PEP for HBV

Of the 400 participants, 134 (32.5%) demonstrated good knowledge of HBV PEP. Approximately half had received prior information about HBV PEP, most commonly through clinical training (56, 27.6%). Fewer than half (174, 43.5%) were aware of the PEP guidelines, and 158 (39.5%) correctly identified the recommended maximum delay for initiating a PEP regimen following exposure (Table 2).

**Table 2:** Knowledge about PEP for HBV among healthcare workers at Wachemo University Nigist Eleni Mohammed Memorial Comprehensive Hospital, 2025.

Questions	Response	Frequency
Have you ever heard of hepatitis B infection PEP	Yes	203 (50.7%)
	No	197 (49.3)
What is the source of information?	Training	56 (27.58%)
	Mass media	34 (16%)
	Text book	32 (15.7%)
	Journals	32 (15.7%)
	Friends	33 (15.8%)
	Others	16 (7.8%)
Have you heard of HBIG?	Yes	90 (27%)
	No	310 (83%)
HBIG can be given intramuscularly (correct)	Yes	202 (50.8%)
	No	198 (49.5%)
HBIG provides short‐term protection against hepatitis B infection. (correct)	Yes	210 (52.5%)
	No	190 (47.5%)
Hepatitis vaccine can be used for PEP (correct)	Yes	100 (25%)
	No	300 (75%)
HBIG used for PEEP (correct)	Yes	211 (52.8%)
	No	189 (47.2%)
The first dose of HBV vaccine/immune globulin should be given as soon as possible after exposure (preferably within 24 h). (correct)	Yes	110 (27.5%)
	No	290 (72.5%)
HBV immunoglobulin (HBIG) is recommended for non‐vaccinated or partially vaccinated exposed individuals. (Correct)	Yes	134 (33.5%)
	No	266 (66.5%)
PEP is necessary after occupational exposure to blood/body fluids. (correct)	Yes	152 (38%)
	No	248 (62%)
Reporting exposures is essential for assessing PEP. (correct)	Yes	190 (47.5%)
	No	210 (52.5%)
Have you had a needle prick in the last 12 months?	Yes	122 (30.5%)
	No	278 (69.5%)
What is the maximum delay for PEP (correct 7 days)	12 h	81 (20.3%)
	48 h	96 (24%)
	72 h	158 (39.5%)
	7days	65 (16.3%)
What is the effectiveness of PEP (correct answer 50–75%)	100%	65 (16.3%)
	75–95%	158 (39.5%)
	50–75%	160 (40%)
	< 50	17 (4.3%)
Have you attained any PEP training	Yes	83 (20%)
	No	317 (80%)
Did you know about the PEP guidelines	Yes	174 (43.5%)
	No	226 (56.5%)

Abbreviations: HBIG=Hepatitis B Immune Globulin, HBV= Hepatitis B virus, PEP= post‐exposure prophylaxis

### 4.3 Knowledge and Associated Factors of Health Care Workers Towards PEP of HBV

In multivariable logistic regression analysis, vaccination status, exposure history, attitude, and prior training were significantly associated with good knowledge of hepatitis B PEP.

HCWs who had been vaccinated against HBV were more likely to have good knowledge compared with unvaccinated HCWs (AOR = 1.07; 95% CI: 1.01–2.86; *p* = 0.04). Those with a history of occupational exposure had higher odds of good knowledge (AOR = 3.72; 95% CI: 2.38–5.80; *p* = 0.002). A positive attitude toward PEP was also strongly associated with good knowledge (AOR = 3.35; 95% CI: 1.95–5.78; *p* = 0.001). Additionally, HCWs who had received PEP training were more likely to have good knowledge (AOR = 2.10; 95% CI: 1.20–3.90; *p* = 0.01).

Sex, age, profession, educational level, years of service, and monthly income were not significantly associated with knowledge after adjustment **(**Table 3 **)**.

**Table 3:** Knowledge and associated factors PEP for HBV among healthcare workers at Wachemo University Nigist Eleni Mohammed Memorial Comprehensive Hospital, 2025.

Variables		Knowledge level		COR (95%CI)	AOR(95%CI)	*p*‐value
		Good (%)	Poor (%)			
**Sex**						
Male		78 (19.5)	155 (38.7)	1.13 (0.72–1.70)	1.92 (0.05–‐2.53)	0.43
Female		52 (13)	115 (28.75)	1	1	
**Age in years**						
20–30		14 (3.5)	17 (4.25)	1	1	
31–40		67 (16.75)	142 (35.5)	1.06 (0.25–4.36)	1.21 (0.1–2.0)	0.51
41–50		49 (12.25)	111 (27.75)	0.50 (0.19–2.52)	0.46 (0.09–2.13)	0.61
**Professions**						
Physician		9 (2.25)	11 (2.75)	1	1	
Nurse		52 (13)	98 (24.5)	4.50 (1.29–17.9)	1.22 (0.26–1.72)	0.75
Laboratory		6 (1.5)	31 (7.75)	1.68 (0.66–4.280	1.82 (0.24–2.78)	0.23
Anesthetist		2 (0.5)	8 (2)	3.68 (0.62–3.01)	1.87 (0.25–‐2.97)	0.54
Midwifery		63 (15)	130 (32.5)	1.54 (0.61–3.95)	1.19 (0.26–1.45)	0.91
**Level of education**						
Diploma		13 (3.25	16 (4)	0.43 (0.13–1.33)	0.23 (0.39–7.3)	0.63
BSC		94 (23.5)	190 (47.5)	0.707 (0.29–1.73)	0.37 (0.51–12.7)	0.76
MSC		16 (4)	44 (11)	0.963 (−0.34 to 2.7)	0.7 (0.81–9.7)	0.59
Physician		7 (1.75)	20 (5)	1	1	
**Year of service**						
6 month to 4 years		91 (22.75)	164 (41)	0.80 (0.2–3.0)	2.0 (0.39–!0)	0.12
5–10years		39 (9.75)	106 (26.5)	0.54 (0.1–2.0)	2.57 (0.5–12)	0.32
> 10 years		3 (0.75)	10 (2.5)	1	1	
**Monthly income**						
> 5000		12 (3)	15 (3.75)	1	1	
5000–10000		699 (17.25)	165 (41.25)	1.30 (0.83–2.1)	1.58 (0.57–4.02)	0.11
> 10000		49 (12.25)	90 (22.5)	0.68 (0.29–1.56)	0.73 (0.42–1.42)	0.33
**Vaccination status**	Yes	96 (23.4)	44 (10.7)	1.43 (1.28–1.67)	1.07 (1.01–2.86)	**0.04**
	No	132 (32.1)	138 (33.6)	1	1	
**Exposure history**	Yes	169 (41.2)	49 (11.9)	3.19 (1.12–1.30)	3.72 (2.38–5.80)	**0.002**
	No	79 (19.2)	122 (29.7)	1	1	
**Attitude**	Positive	111 (27.75)	169 (42.25)	1.60 (1.28–.9.2)	3.35 (1.95‐–5.78)	**0.001**
	Negative	19 (4.75)	101 (25.25)	1	1	
**Ever taking training**	Yes	39 (9.7)	44 (11)	1.10 (1.43–3.83)	2.1 (1.2–3.9)	**0.01**
	No	99 (24.7)	218 (54.5)	1	1	

*Note: p* < 0.05 (bolded) indicates a statistically significant association

Abbreviations: AOR = adjusted odds ratio, CI = confidence interval, COR = crude odds ratio, HBV = Hepatitis B virus = PEP, post‐exposure prophylaxis

### 4.4 Attitude of HCWs about PEP for HBV

More than half, 280 (70%) of the study participants had a positive attitude toward PEP of HBV. The majority of study respondents, 322 (80.5%), 352 (88%), and 289(72.2%), agreed on the importance of training for behavior changes, and PEP is indicated for all sharp injuries, respectively. However, only 63 (16.5%) trust PEP effectiveness (Table 4).

**Table 4:** Attitude about PEP for HBV among healthcare workers’ knowledge about PEP for HBV among health care workers at Wachemo University Nigist Eleni Mohammed Memorial Comprehensive Hospital, 2025.

Questions	Response	Frequency
Do you think PEP is important?	Yes	272 (68%)
	No	128 (32%)
Do you believe that training of PEP is important for a behavioral change	Agree	322 (80.5%)
	Disagree	78 (19.5%)
	Neutral	0
Do you think there should be a PEP guideline in the workplace	Agree	289 (72.3%)
	Dis agree	65 (16.3)
	Neutral	46 (11.3%)
Do you believe PEP helps to prevent further HBV infection?	Agree	192 (48%)
	Disagree	145 (36.3%)
	Neutral	63 (15.7%)
What is your opinion on the saying that PEP is indicated for any type of sharp injury?	Agree	352 (88%)
	Dis agree	32 (8%)
	Neutral	66 (16.5%)
What is your opinion on the belief that PEP is not important if the exposure is not with the patient's blood of known HBV positivity?	Agree	352 (88%)
	Disagree	32 (8%)
	Neutral	66 (16.5%)
HCWs start PEP even if not willing.	Agree	228 (57%%)
	Disagree	100 (25%)
	Neutral	2 (0.5%)
Occupational exposure to HBV is avoidable.	Agree	200 (50%)
	Disagree	0
	Neutral	200 (50%)

Abbreviations: HBV = Hepatitis B virus, PEP = post‐exposure prophylaxis

### 4.5 Practice of HCWs about PEP for HBV

Less than half of the participants were vaccinated against HBV (44.5%). Ninety respondents (22.5%) reported exposure to high‐risk situations, of whom 35 (38.2%) sustained a needle stick injury. Only 20 (22.2%) of the exposed respondents received PEP, while the majority (70, 77.8%) did not. Among those who received PEP, 18 (90.0%) had been exposed to blood from known HBV‐positive patients, and 2 (10.0%) were injured by sharp objects or exposed to blood from patients with unknown HBV status. The most commonly reported reason for not receiving PEP was a lack of awareness of the availability of PEP services and protocols (50, 71.5%)(Table 5).

**Table 5:** Practice about PEP for HBV among health care workers at Wachemo University Nigist Eleni Mohammed Memorial Comprehensive Hospital.

Questions	Response	Frequency (%)
Have you ever been vaccinated against HBV	Yes	178 (44.5%)
	No	222 (55.5%)
	I don't remember	
Ever been exposed to HBV in risky conditions	Yes	90 (22.5%)
	No	310 (77.5)
	I don't remember	0
Took PEP after exposure	Yes	20 (22.2%)
	No	70 (77.8%)
Reasons for taking PEP after exposure	Exposure to blood from known HBV‐positive patients	18 (90%)
	Exposure to blood from patients with unknown HBV status	1 (5%)
	Injury from the sharp object	1 (5%)
	Contact patients with body fluids.	
Reasons for not taking PEP	Unaware of the existence of the PEP service and protocol	50 (71.5%)
	Lack of understanding of the value of reporting exposure	
	Fear, stigma, and discrimination	
	PEP service unavailable	20 (28.5%)
	Client tested negative	
What time did you start taking PEP	Within 1 h	
	After 2–‐12 h	
	12–48 h	15 (75%)
	48–72 h	5 (25%)
	72 h to 7 days	
Have you reported the occurrence of injury	Yes	35 (38.8%)
	No	65 (61.2%)
Have you tested/checked after the course of treatment	Yes	2 (10%)
	No	18 (90%)

*Note: N* = 90(denominator) number of exposed study participants.

Abbreviations: HBV = Hepatitis B virus, PEP = post exposure prophylaxis

## 5 Discussion

This study evaluated the knowledge, attitude, and practice (KAP) of healthcare workers regarding hepatitis B virus (HBV) post‐exposure prophylaxis (PEP) and identified factors associated with good knowledge. The findings demonstrate considerable deficiencies in knowledge and practice, despite generally positive attitudes toward HBV PEP. To date, evidence on this topic in Ethiopia remains limited, with no prior studies comprehensively assessing HBV PEP–related KAP among healthcare workers, despite the country's high HBV burden and frequent occupational exposures. Strengthening healthcare workers’ KAP on HBV PEP should therefore be prioritized as a public health intervention. Key strategies include regular in‐service training, pre‐employment and catch‐up HBV vaccination programs, establishment of accessible and timely PEP services, and periodic monitoring of KAP to guide targeted interventions [23].

Hepatitis B virus (HBV) infection remains a significant occupational hazard for healthcare workers (HCWs), particularly in low‐income countries where the burden of the disease is high, and vaccination coverage may be suboptimal. Post‐exposure prophylaxis (PEP) plays a critical role in preventing HBV infection following occupational exposure to potentially infectious blood or body fluids. The effectiveness of PEP, however, is strongly influenced by the level of knowledge, the attitude towards its importance, and the practice of prompt and appropriate measures after exposure [24, 25].

Healthcare personnel are recognized to be constantly at risk of occupational exposure to body fluids, including blood, which can spread HBV and other blood‐borne viruses [26]. Thus, it should be a top priority for all healthcare facilities to improve the occupational health and safety of healthcare workers by improving their knowledge of PEP for HBV [23]. Accordingly, our study assessed the knowledge of PEP for HBV, which is an important strategy for the prevention of occupational acquisition of HBV among HCWs. Accordingly, our study assessed the knowledge of PEP for HBV, an important strategy for preventing occupational acquisition of HBV among HCWs. The findings revealed inadequate knowledge, with an overall mean score of 32.5%. This poor level of knowledge might primarily be attributed to a lack of training on PEP and limited awareness of its availability.

Our study demonstrated that healthcare workers (HCWs) had limited knowledge about post‐exposure prophylaxis (PEP) for hepatitis B infection. Fewer than one‐third of participants were aware that PEP even exists, which indicates a significant knowledge gap. This gap may be partly attributed to the lack of structured training on hepatitis B prevention and management. The Ethiopian National Policy on PEP highlights training of healthcare professionals as a key strategy for ensuring appropriate post‐exposure management [27]. However, in our study, only 27.6% of HCWs reported receiving training that enhanced their awareness of hepatitis B PEP. The absence of adequate training not only restricts individual awareness but also limits opportunities for peer‐to‐peer information sharing, thereby perpetuating low knowledge levels across the workforce [28]. As a result, many HCWs remain unaware of their occupational risk of acquiring hepatitis B and are left vulnerable despite the availability of effective PEP measures.

Comparable findings have been documented in other sub‐Saharan African countries, such as Ghana and Uganda, where only 12.1% of HCWs were aware of hepatitis B immunoglobulin (HBIG) and the hepatitis B vaccine as available PEP options [20, 29]. This low level of awareness mirrors the findings of our study and highlights a broader regional challenge in ensuring that HCWs are adequately informed about evidence‐based post‐exposure management strategies. The similarity of results across different countries suggests that insufficient training and limited access to continuous professional development may be systemic issues within healthcare systems in the region. Furthermore, the lack of awareness about effective PEP options is particularly concerning given the high occupational risk of hepatitis B among HCWs in resource‐limited settings, where safe injection practices, proper disposal of sharps, and consistent use of protective equipment are not always guaranteed. These findings underscore the urgent need for targeted educational interventions and integration of hepatitis B prevention strategies into existing infection prevention and control programs to improve both knowledge and practice among HCWs.

The administration of HBIG provides the main protection after exposure to hepatitis B among individuals who do not respond to hepatitis B vaccination or among people who have not received vaccination [30]. However, this was less known by the HCWs in our study. More than a third of HCWs were unaware of HBIG. In addition, only 25% of the HCPs were aware that the hepatitis B vaccine could be used as a hepatitis B PEP. Awareness about HBIG as a PEP option was higher in this study than in a study in Ghana (2.8%) [29]. Therefore, these results underline the necessity of informing healthcare workers about the various hepatitis B PEP alternatives, which are crucial steps in reducing the risk of acquiring the virus at the moment of exposure.

It is concerning that the current study found an inadequate level of knowledge about hepatitis B PEP, given the significant possibility of hepatitis B infection that exists in healthcare settings. Given the significant risk of blood‐borne infections, over 30.5% of the healthcare professionals in our study reported having experienced needle stick injuries in the previous 12 months. Without access to PEP and behavioral modification, a sizable percentage of exposures may result in hepatitis B infections in HCWs, which would ultimately affect the health of HCWs and their delivery of healthcare services [15, 31].

Our study revealed that the majority of healthcare workers were not vaccinated against the hepatitis B virus (HBV), which raises serious concerns given their occupational risk of exposure. The vaccination coverage observed in our study was lower than that reported in a previous study conducted in Karachi, Pakistan [32]. This difference may partly be explained by the absence of a policy in our study setting mandating HBV vaccination for healthcare workers. In contrast, countries with stronger occupational health policies and institutional vaccination programs have reported higher uptake rates, demonstrating the critical role of structured policies in improving vaccine coverage. The lack of a mandatory vaccination policy not only leaves HCWs vulnerable but also increases the risk of nosocomial transmission to patients, colleagues, and the wider community. Moreover, poor vaccination coverage may reflect systemic barriers such as limited vaccine availability, lack of awareness of HBV risks, and financial constraints that hinder voluntary uptake. These findings emphasize the urgent need to establish clear institutional and national policies that require HBV vaccination for all HCWs, alongside accessible vaccination programs and regular monitoring to ensure compliance. Implementing such measures could substantially reduce HBV transmission risk and strengthen infection prevention and control practices in healthcare facilities.

Healthcare workers exhibited a positive attitude towards the PEP of HBV. Over 70% of participants agreed on the importance of PEP for HBV and the availability of PEP guidelines in the workplace. This study is comparable in Nigeria. However, the PEP practice of HBV was low. This result tends to support the idea that PEP for HBV has to be improved in the study area [33]. This could probably be a lack of a PEP training center.

In this study, a significant difference was observed regarding whether healthcare workers had received training in PEP. This finding highlights the need to ensure that all categories of HCWs are adequately supported with training and education on the WHO‐recommended protocols for managing occupational exposure to blood and body fluids infected with HBV. Providing standardized and accessible training opportunities could help bridge existing gaps and promote uniform preparedness across different professional groups. Interestingly, despite differences in training, no significant variation in knowledge levels was found between the different HCW categories. This contrasts with the findings of a study conducted in Ghana [34]. where differences in knowledge were reported among professional groups. The discrepancy may be explained by variations in sample size, as our study included a larger number of participants, which might have minimized differences between groups. These observations suggest that while training is an essential component in strengthening PEP practice, broader strategies such as continuous professional development and institutional policies are equally important to achieve consistent knowledge and practice across the healthcare sector.

## 6 Conclusion and Recommendations

The study reveals significant knowledge and practice gaps among HCPs regarding PEP for the hepatitis B virus, although the majority demonstrated positive attitudes towards it. Most healthcare workers were at risk of HBV; however, only a small percentage utilized PEP, mainly because they were unfamiliar with the availability of PEP services and protocols. To address these gaps, the study highlights the need for context‐specific educational programs on the hepatitis B virus and other emerging infectious diseases of public health concern. Such initiatives should be tailored to bridge the identified knowledge gaps, with a particular focus on younger healthcare providers and those without prior training. Furthermore, continuous professional development should be prioritized through regular workshops, seminars, and online courses to ensure that all healthcare professionals remain updated on the latest information about hepatitis B vaccination and PEP. Governments and policymakers should also ensure universal coverage by providing free or subsidized vaccination for all healthcare workers, students in health sciences, and high‐risk populations. In addition, mass media, community outreach, and social media platforms should be utilized to raise awareness about hepatitis B and PEP.

## Author Contributions

**Esubalew Muluneh Aligaz** and **Zekarias Markos:** took part in conceptualization, methodology, formal analysis, investigation, resources, data curation, writing–original manuscript draft, writing–review and editing, visualization, and supervision. **Sitotaw Tesfa Zegeye** and **Samuel Debas Bayable:** took part in methodology, formal analysis, investigation, writing review and editing, and visualization manuscript writing, paper revision, and editing. Esubalew Muluneh Aligaz is a guarantor for the overall content.

## Ethics Statement

The study was conducted in accordance with the Helsinki Declaration, which outlines ethical principles for medical research involving human participants. Approval was granted by the ethical review committee at Wachemo University, Nigist Eleni Mohammed Memorial Comprehensive Hospital in 2025, with the protocol number Ref: WCMH 10/2025. edu. et.

## Consent

Written informed consent was obtained from all participants. Throughout the study, privacy and confidentiality were upheld by removing any personal identifiers.

## Funding

The authors received no specific funding for this work.

## Conflicts of Interest

The authors declare no conflicts of interest.

## Transparency Statement

The lead author, Esubalew Muluneh Aligaz, affirms that this manuscript is an honest, accurate, and transparent account of the study being reported; that no important aspects of the study have been omitted; and that any discrepancies from the study as planned (and, if relevant, registered) have been explained.

## Data Availability

The datasets used and analyzed during the current study are available from the corresponding authors upon reasonable request.
